# A Rare Presentation of a Pilar (Trichilemmal) Cyst of the Upper Arm in an Elderly Male

**DOI:** 10.7759/cureus.115504

**Published:** 2026-08-31

**Authors:** Jasndeep Kaler, Ulrica S Armbrister, Taylor Tomko, Frederick Tiesenga

**Affiliations:** 1 Surgery, Windsor University School of Medicine, Cayon, KNA; 2 Surgery, American University of Barbados, Bridgetown, BRB; 3 General Surgery, West Suburban Medical Center, Chicago, USA

**Keywords:** adnexal tumors, benign cutaneous cyst, pilar cyst, proliferating trichilemmal tumor, surgery general, surgical case reports, surgical excision, trichilemmal cyst, upper arm mass

## Abstract

Pilar cysts, also known as trichilemmal cysts, are benign keratin-filled lesions that arise from the outer root sheath of the hair follicle. Pilar cysts account for a small minority of cutaneous cysts and are most commonly found on the scalp, particularly in middle-aged women. Because they overwhelmingly occur on the scalp, presentation in other anatomical locations is uncommon and can create diagnostic uncertainty. We present the case of an 81-year-old male with a slowly enlarging, painless mass on the left upper arm that had been present for nearly eight years. The lesion was well-circumscribed and firm on examination, without associated erythema, drainage, or tenderness. Given its gradual growth, surgical excision was performed. Grossly, the mass was encapsulated and measured approximately 1.5 cm in diameter. Histopathologic evaluation demonstrated a keratin-filled cyst lined by stratified squamous epithelium with abrupt trichilemmal keratinization and absence of a granular layer, confirming the diagnosis of a benign pilar cyst. There was no evidence of proliferative or malignant transformation. The patient tolerated the procedure well and had an uncomplicated postoperative course. Although pilar cysts are benign, their presence in atypical locations such as the upper extremity may broaden the differential diagnosis to include epidermoid cysts, lipomas, and other soft tissue neoplasms. This case highlights the importance of maintaining clinical suspicion for pilar cysts outside of the scalp and emphasizes the role of histopathologic confirmation in establishing an accurate diagnosis. Recognizing these rare presentations can help prevent misdiagnosis and ensure appropriate surgical management.

## Introduction

Cysts derived from the hair follicle apparatus constitute a notable subset of benign cutaneous lesions, among which the pilar cyst is well recognized. Typically, pilar cysts arise from the outer root sheath of the hair follicle, specifically in the isthmic region. They affect 5-10% of the population and are most frequently observed on the scalp of middle-aged to older adults, predominantly in women [1]. Histologically, these cysts are distinguished by abrupt keratinization, meaning they lack a granular cell layer and often contain homogeneous keratin material rather than the lamellated keratin and granular layer typical of epidermal cysts [2].

The occurrence of a pilar cyst in the upper arm represents an atypical presentation. Although hair follicles are present in this region, pilar cysts show a marked predilection for the scalp, where terminal hair follicles are abundant, and the follicular outer root sheath from which these cysts originate is well developed. In contrast, the upper arm has a lower density of terminal hair follicles and a greater proportion of finer vellus hairs, providing fewer follicular structures typically associated with trichilemmal cyst formation. Nevertheless, because pilar cysts arise through trichilemmal keratinization of the outer root sheath, they may theoretically develop at any hair-bearing site containing the appropriate follicular epithelium. Their occurrence in the upper arm is therefore anatomically plausible but uncommon. Recent case reports have documented pilar cysts in unusual locations, such as the dorsum of the hand, underscoring the need for awareness of such variants in clinical and pathological differential diagnosis [3].

Although pilar cysts are benign, rare progression to proliferating trichilemmal tumors (PTTs) has been described, making histopathologic evaluation important for excluding atypical or malignant features [4]. At an uncommon site such as the upper arm, the clinical differential diagnosis may include epidermal cysts, lipomas, pilomatricomas, and other soft-tissue neoplasms. Histopathologic confirmation is therefore particularly important in establishing the correct diagnosis and distinguishing a benign pilar cyst from other cutaneous and subcutaneous lesions.

Given this background, the present case aims to characterize the clinical and histopathologic features of a pilar cyst arising in the upper arm and to highlight the diagnostic and management considerations associated with this uncommon anatomical presentation.

## Case presentation

An 81-year-old male presented with a slowly enlarging mass located on the proximal posterior aspect of the left upper arm. The patient first noted the lesion approximately eight years prior and reported a gradual increase in size over time. The mass was largely asymptomatic, without pain, erythema, or drainage, although the patient reported occasional pruritus in the area. On physical examination, the lesion was firm, fixed, and subcutaneous, without overlying skin changes. The size of the lesion at the preoperative examination was not documented. A similar lesion in the right posterior auricular region had been excised one year earlier but was not submitted for histopathologic evaluation.

The patient’s past medical history was significant for coronary artery disease status post percutaneous coronary intervention with drug-eluting stent placement following a myocardial infarction, hypertension, type 2 diabetes mellitus, dyslipidemia, benign prostatic hyperplasia, and moderate obesity. Past surgical history included coronary angioplasty with stent placement in 2005, excision of a reportedly benign facial skin mass in 2022-2023, for which histopathologic examination details were unavailable, and an unspecified neck surgery in July 2024, which the patient believed to involve gland removal.

The patient had no known drug allergies. Home medications included aspirin 81 mg daily, carvedilol 25 mg, glipizide 5 mg, losartan 100 mg, metformin 1000 mg, rosuvastatin 40 mg, and tamsulosin 0.4 mg. He was a former smoker with a three-year history who quit over 16 years ago and denied alcohol or illicit drug use. Family history was notable for malignant neoplastic disease in his mother, hypertension in his father, lung cancer in his aunt, and malignant neoplasm of the lower limb (knee) in his daughter.

Based on the clinical appearance and subcutaneous location of the lesion, the preoperative impression was a lipoma or other benign soft-tissue mass. The clinical differential diagnosis included an epidermal cyst, pilar cyst, lipoma, pilomatricoma, and other soft-tissue neoplasms. No preoperative imaging was documented. Given the longstanding and progressively enlarging nature of the lesion, its clinical appearance, and the patient's history of a previously excised similar lesion, preoperative biopsy was not pursued, and complete surgical excision was considered appropriate for both diagnostic and therapeutic purposes.

Routine preoperative evaluation was completed, and the patient was medically cleared for surgery. On September 9, 2025, he underwent excision of the left upper arm subcutaneous mass. The patient was prepped and draped in standard sterile fashion. A direct incision was made over the lesion, and an approximately 2 × 3 cm area was excised down to the deep subcutaneous tissue, as seen in Figure 1.

**Figure 1:**
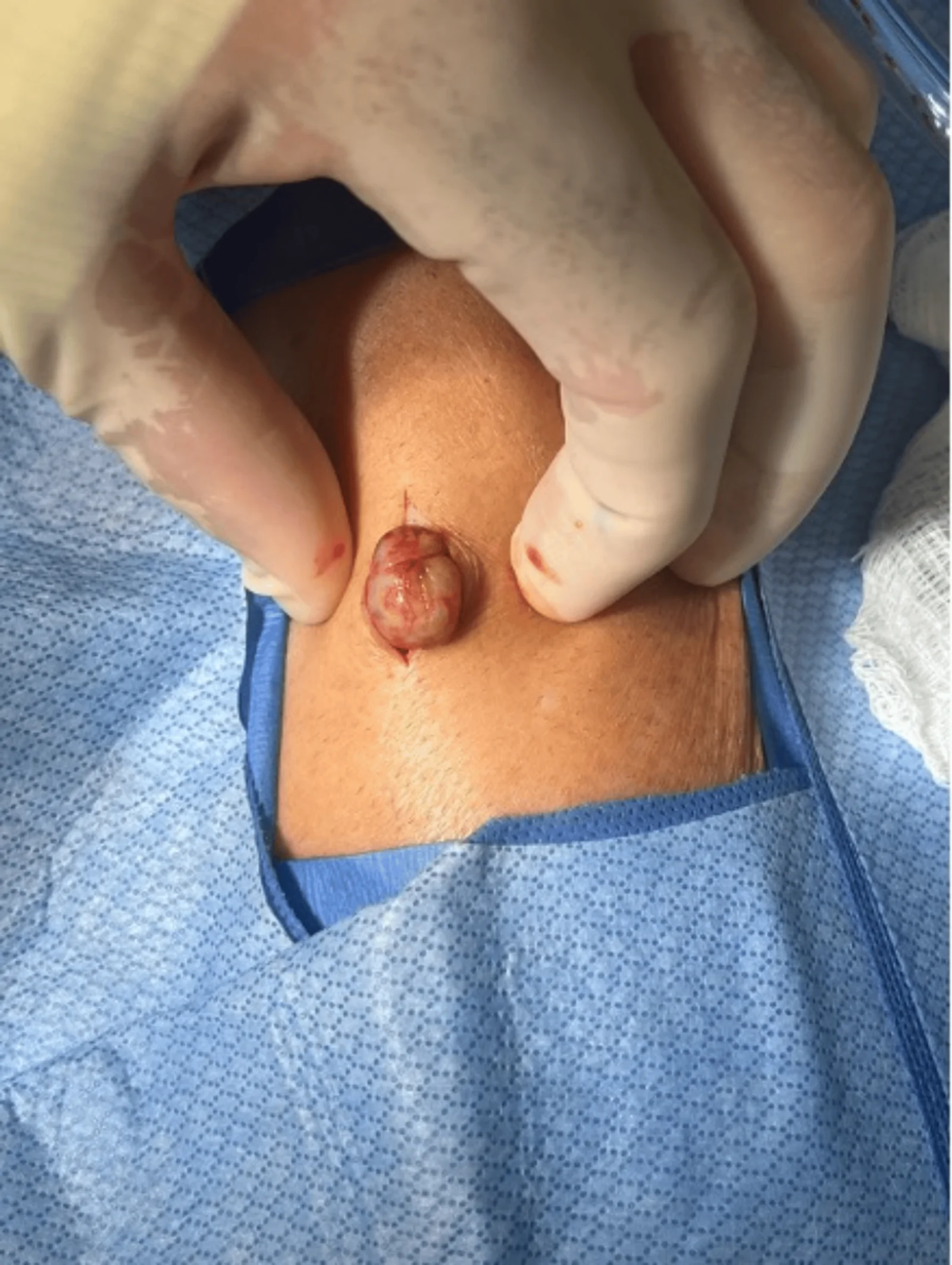
Intraoperative photograph showing a well-circumscribed, firm, subcutaneous pilar cyst on the left upper arm following incision and exposure of the lesion.

The excised specimen, as visible in Figure 2, was sent for histopathologic analysis.

**Figure 2:**
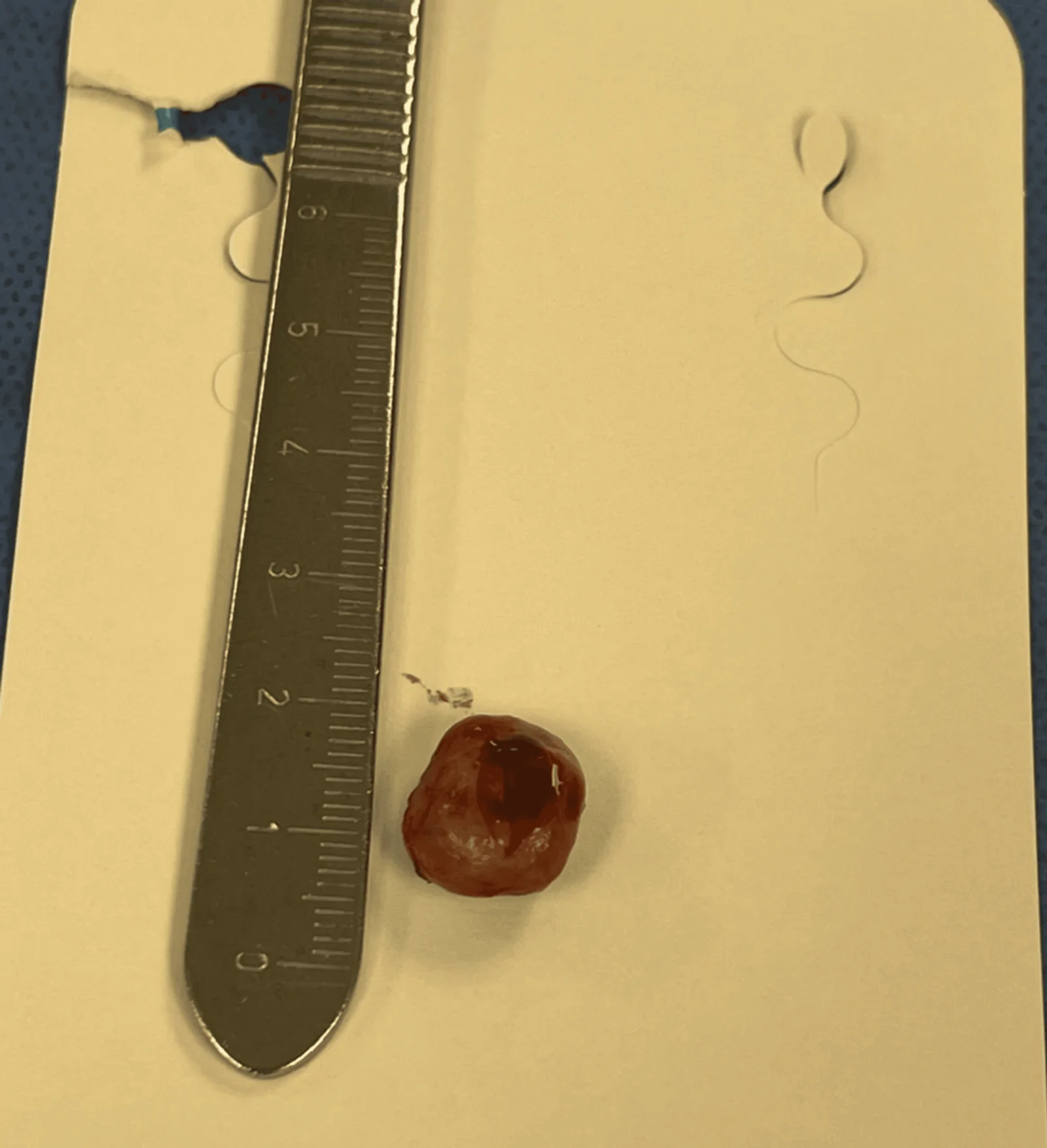
Gross specimen of the excised pilar cyst following complete removal, measuring about 1.5 cm in diameter. The cyst wall is intact, and the lesion appears well-encapsulated, typical of pilar cyst morphology.

The patient tolerated the procedure well and was discharged home the same day. The pathology report confirmed a benign pilar cyst with no evidence of malignancy, as displayed in Figure 3. Complete excision was achieved intraoperatively. The patient was advised to follow up as needed; however, no subsequent clinical follow-up was documented, and therefore, the long-term postoperative status and recurrence of the lesion could not be assessed.

**Figure 3:**
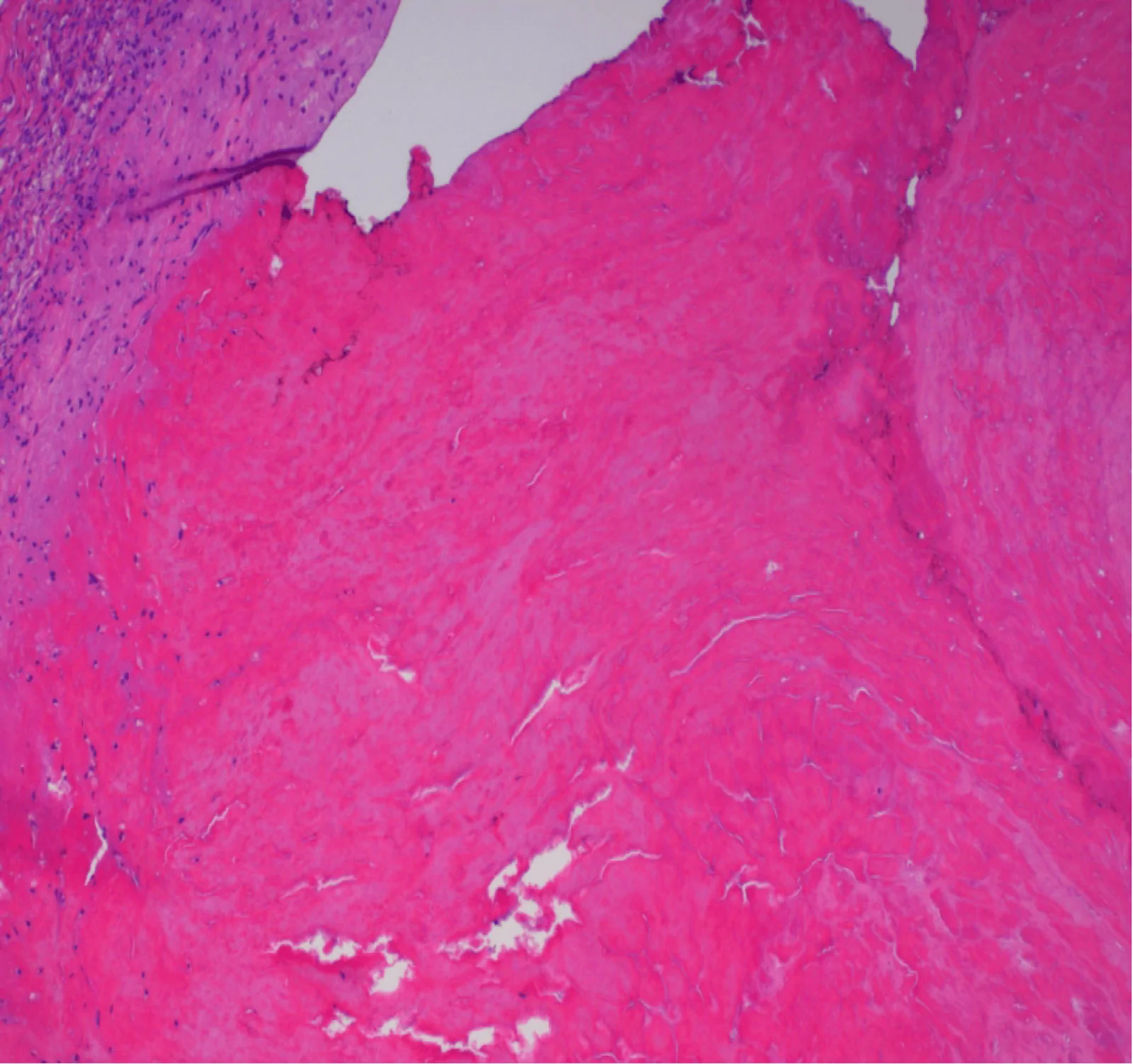
Histopathologic image (H&E stain) demonstrating a keratin-filled cyst lined by stratified squamous epithelium with abrupt trichilemmal keratinization, characteristic of a benign pilar cyst. The cyst wall is composed of stratified squamous epithelium with surrounding fibrous connective tissue. No malignant features were identified. Calcification and mitotic activity were not specifically described in the histopathology report.

## Discussion

Pilar cysts, also known as trichilemmal cysts, are benign keratin-filled lesions that arise from outer root sheath differentiation and characteristically demonstrate abrupt trichilemmal keratinization without a granular layer on histology. They occur predominantly on the scalp, representing up to 90% of cases, and are rarely found in extracranial locations, making involvement of the upper arm an uncommon presentation [5].

These cysts show a clear female predominance and typically present in middle-aged adults. While most are sporadic, familial trichilemmal cysts have been described with autosomal dominant inheritance. Recent genetic studies have identified pathogenic variants in the PLCD1 gene as a key mechanism for hereditary disease, supporting a monoallelic two-hit model involving a germline and a somatic mutation on the same allele [6-8]. Clinical indicators of a hereditary tendency include multiple cysts, scalp location, and a positive family history [5].

Although simple pilar cysts are benign, PTTs and malignant PTTs (MPTTs) can rarely develop, particularly in long-standing or recurrent lesions. Risk factors associated with malignant transformation include advanced age, rapid enlargement, ulceration, and repeated trauma [9-11]. Immunosuppression has also been described as a possible cofactor in isolated cases of trichilemmal carcinoma [12].

At atypical sites such as the extremities, pilar cysts are frequently misdiagnosed as epidermoid cysts, lipomas, or other adnexal neoplasms. Histopathologic evaluation remains the diagnostic gold standard, revealing abrupt keratinization without a granular layer and a cyst wall resembling the outer root sheath of the hair follicle [13]. Proliferating variants show lobulated squamous proliferation that may mimic squamous cell carcinoma both clinically and microscopically [9,11]. Imaging is seldom required but can help delineate the extent of large or deep lesions [5].

Complete surgical excision, including the cyst capsule, remains the treatment of choice to prevent recurrence. Incomplete excision is the primary cause of recurrence, with reported rates ranging from 3% to 10% depending on the adequacy of removal and the presence of proliferative components [14]. Histopathologic confirmation is essential to rule out proliferative or malignant change. For proliferating or malignant variants, wide local excision with histologic margin control is recommended, as local recurrence and rare metastases have been documented [9,11]. Regular clinical follow-up is advised, particularly for lesions demonstrating atypical histologic features or incomplete initial removal.

The present case of an 81-year-old male with a pilar cyst on the left upper arm highlights an uncommon extracranial presentation of a trichilemmal cyst, particularly involving the upper limb. Pilar cysts arising at unusual upper-extremity sites have previously been described, including lesions on the dorsum of the hand [15,16]. Recognition of this uncommon presentation broadens awareness of the anatomic distribution of these lesions and underscores the importance of considering pilar cysts in the differential diagnosis of subcutaneous nodules in unusual locations. Proper identification and complete excision can minimize the risk of recurrence and prevent diagnostic confusion with more aggressive cutaneous neoplasms.

## Conclusions

Pilar cysts are benign adnexal lesions that most commonly arise on the scalp, making their occurrence at extra-scalp sites such as the upper arm uncommon. This case describes a pilar cyst arising on the proximal posterior upper arm of an elderly male and highlights the importance of including pilar cysts in the differential diagnosis of subcutaneous lesions occurring at atypical anatomical sites. Histopathologic examination remains important for establishing the definitive diagnosis and distinguishing a benign pilar cyst from other cutaneous and subcutaneous lesions with similar clinical presentations. Complete surgical excision allows both definitive histopathologic evaluation and treatment; however, because no subsequent clinical follow-up was documented in this case, long-term postoperative status and recurrence could not be assessed.
